# HBOT-induced noncardiogenic pulmonary edema in acute carbon monoxide poisoning: A case report and mechanistic insights

**DOI:** 10.1097/MD.0000000000044925

**Published:** 2025-09-26

**Authors:** Min-Xue Yao, Yu-Yang Chen, Zhi-Jian Gu

**Affiliations:** a Department of Emergency Medicine, Affiliated Kunshan Hospital of Jiangsu University, Kunshan, Jiangsu Province, China.

**Keywords:** acute carbon monoxide poisoning, acute pulmonary edema, case report, hyperbaric oxygen therapy, patient prognosis

## Abstract

**Rationale:**

Acute carbon monoxide poisoning is a common medical emergency, and hyperbaric oxygen therapy (HBOT) serves as the cornerstone of treatment. Although rare, serious complications such as acute pulmonary edema (APE) can be life-threatening. Most reported cases involve patients with preexisting cardiac conditions, making this case of APE in a patient without cardiac comorbidities particularly noteworthy.

**Patient concerns:**

A 35-year-old male patient developed dyspnea accompanied by severe coughing during the decompression phase of HBOT for acute carbon monoxide poisoning.

**Diagnoses:**

Emergency chest computed tomography showed APE.

**Interventions:**

Immediate endotracheal intubation and mechanical ventilation were initiated, along with a combination of diuretics, glucocorticoids, and bronchodilators.

**Outcomes:**

During the 3-month follow-up period, the patient remained asymptomatic with complete resolution of clinical manifestations.

**Lessons:**

Although HBOT-induced APE is a rare complication, its critical clinical implications necessitate rigorous monitoring of respiratory dynamics and oxygenation status during treatment sessions.

## 1. Introduction

Hyperbaric oxygen therapy (HBOT), which accelerates the dissociation of carbon monoxide (CO) from hemoglobin and facilitates its elimination to prevent delayed neurological sequelae, is regarded as a primary interventions for acute carbon monoxide poisoning (ACOP).^[1]^ Recent meta-analyses suggest that HBOT can reduce the incidence of delayed neurological sequelae compared with normobaric oxygen alone.^[2]^ Although HBOT has a favorable safety profile overall, with serious adverse effects being rare, minor side effects such as barotrauma are relatively common. This report describes an uncommon case of HBOT-induced acute pulmonary edema (APE), emphasizing the need for vigilance regarding its potential risks even in patients without cardiac comorbidities.

## 2. Case report

A 35‐year‐old male with no significant past medical history – including no history of asthma, heart failure, or allergies to food or medications – was found unconscious at approximately 7:00 am on December 24, 2024, by his wife. The incident occurred in a closed room containing a burned-out charcoal brazier. His wife promptly contacted emergency services, and upon arrival, the attending physician administered oxygen via a face mask before transporting him to the emergency department.

**Figure 1.**
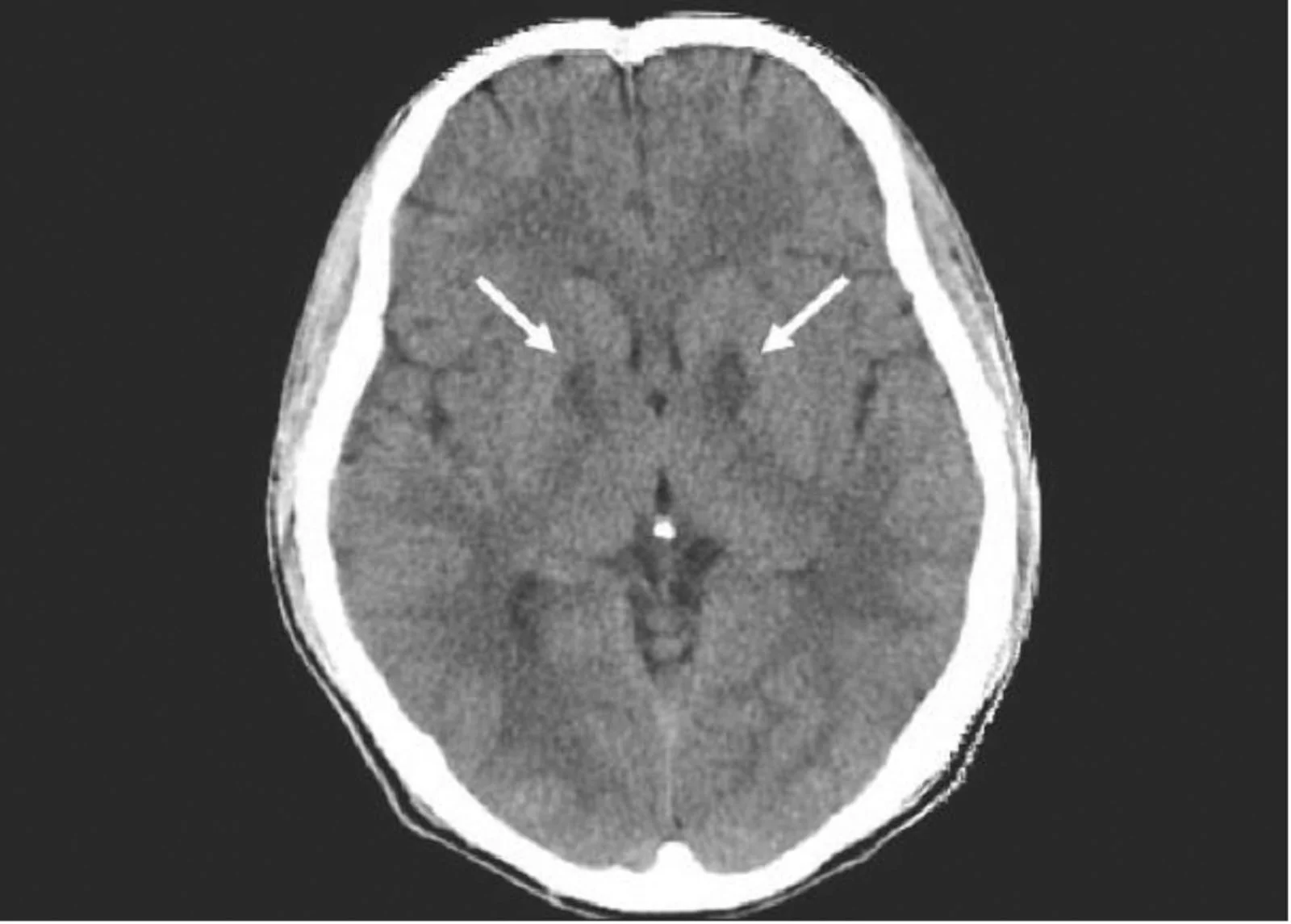
CT performed at the time of admission demonstrated hypodense lesions within the bilateral globus pallidus. CT = computed tomography.

On arrival at the emergency department, his vital signs were as follows: temperature 37.2°C, heart rate 115 beats/min, respiratory rate 30 breaths/min, oxygen saturation 91%, and a Glasgow Coma Scale score of E2V2M5. Arterial blood gas analysis revealed a pH of 7.34, partial pressure of oxygen 60.3 mm Hg, partial pressure of carbon dioxide 34.1 mm Hg, lactate 5.3 mmol/L, bicarbonate 18.4 mmol/L, and carboxyhemoglobin 23%. Additional laboratory tests indicated a white blood cell count 14.82 × 10⁹/L with neutrophils comprising 86.2%, alanine aminotransferase 136 U/L, aspartate aminotransferase 173 U/L, blood urea nitrogen 9.72 mmol/L, creatinine 94.2 μmol/L, troponin I 0.38 ng/mL, myoglobin > 900 ng/mL, and procalcitonin 2.1 ng/mL. A 12‐lead electrocardiogram demonstrated sinus tachycardia without evidence of acute ischemia or infarction. Computed tomography (CT) showed reduced density in the bilateral globus pallidus (Fig. 1), without notable pulmonary abnormalities.

**Figure 2.**
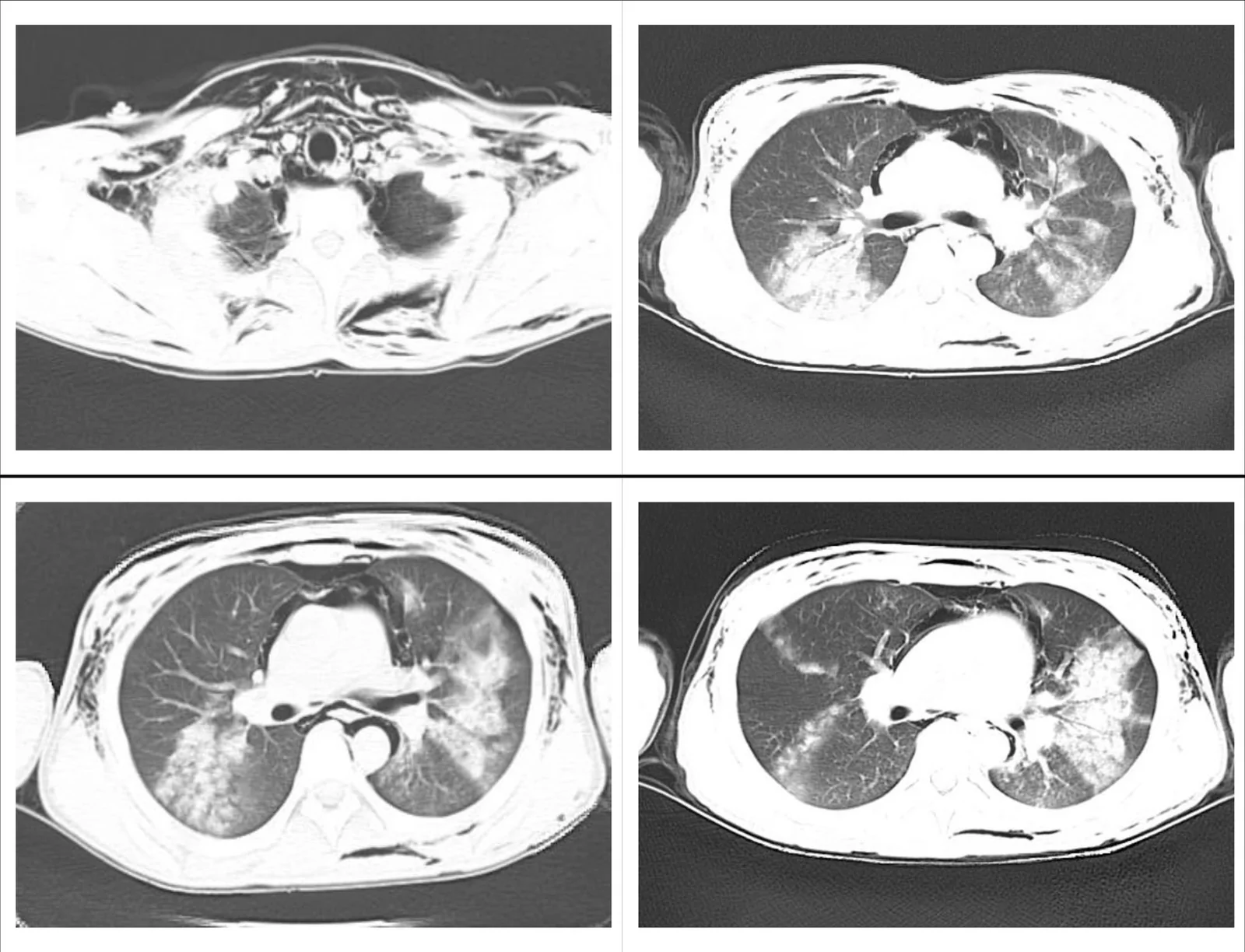
CT performed after HBOT demonstrated bilateral pulmonary exudative changes, subcutaneous emphysema, pneumomediastinum, and pneumothorax. CT = computed tomography, HBOT = hyperbaric oxygen therapy.

**Figure 3.**
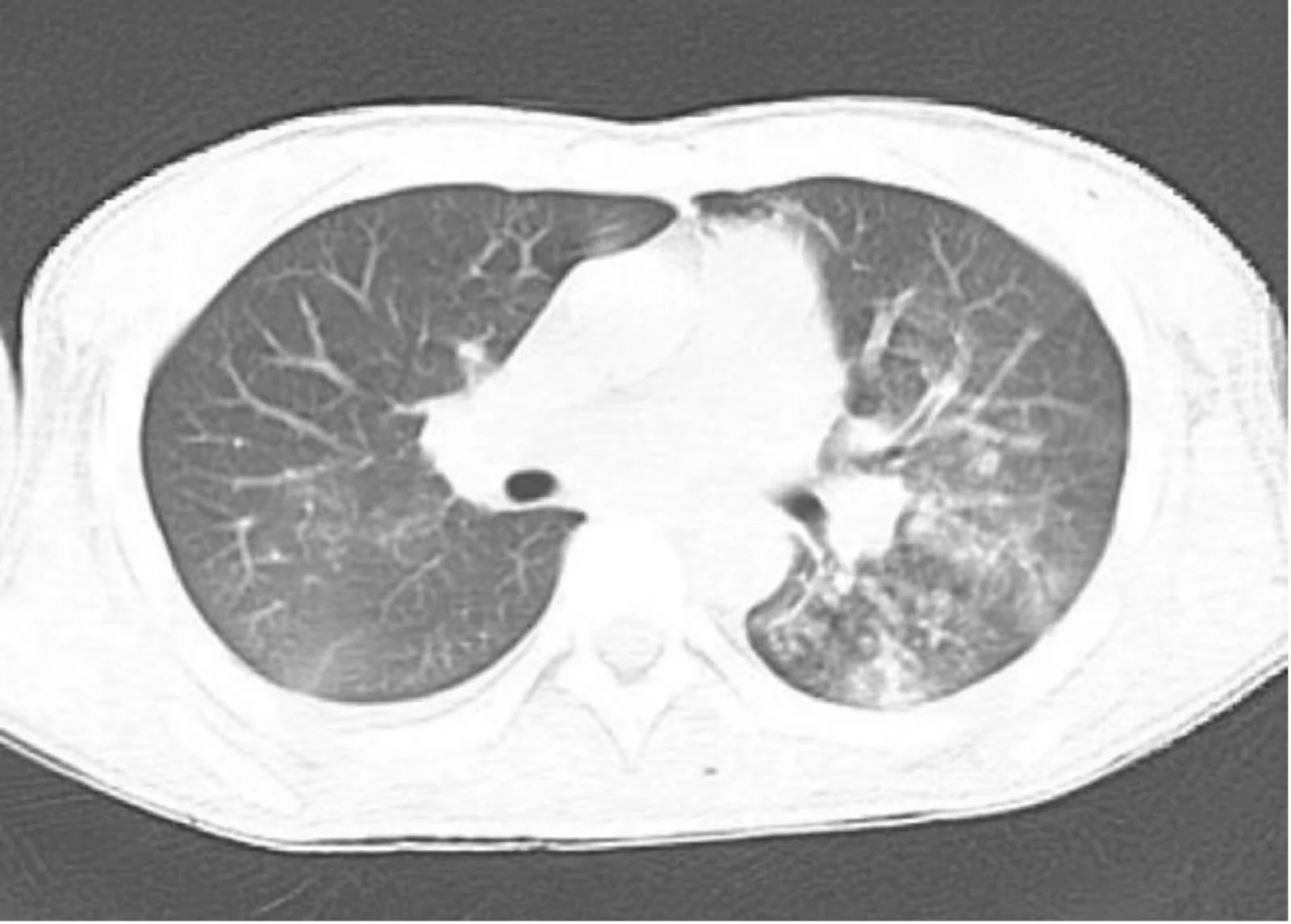
A repeat CT performed 5 days after treatment demonstrated significant improvement in pulmonary edema with complete resolution of the subcutaneous, mediastinal, and subpleural gases. CT = computed tomography.

Given the patient’s critical condition, we initiated emergency HBOT with a protocol of 100% oxygen at 0.25 MPa for 60 minutes, followed by decompression at a rate of 0.01 MPa/min. During the decompression phase, the patient suddenly developed dyspnea accompanied by a severe cough and expectoration of frothy sputum. Upon admission to the ward, his heart rate increased to 135 beats/min, blood pressure was 161/103 mm Hg, respiratory rate increased to 38 breaths/min, and oxygen saturation decreased to 82%. Auscultation revealed coarse bilateral crackles and expiratory wheezing across all lung fields. Palpation detected crepitus extending from the anterior chest wall to the supraclavicular regions. These findings are indicative of APE complicated by subcutaneous emphysema, with a clinical suspicion of possible pneumothorax. Immediate interventions included endotracheal intubation, mechanical ventilation, and administration of diuretics, corticosteroids, and bronchodilators. Once the patient’s vital signs stabilized, a follow-up pulmonary CT examination was performed, which revealed diffuse pulmonary exudation, mediastinal emphysema, a small bilateral pneumothorax, and subcutaneous emphysema in the chest, abdominal, and cervical root regions (Fig. 2). Transthoracic echocardiography performed at rest showed no significant abnormalities. Following active treatment, the patient’s condition gradually stabilized, and the endotracheal tube was removed after 48 hours. A repeat CT scan demonstrated significant improvement in pulmonary edema with complete resolution of the subcutaneous, mediastinal, and subpleural gases (Fig. 3). The patient was subsequently transferred to the Department of Rehabilitation Medicine for further care and discharged in a recovered state 10 days later. At the 3-month follow-up, he was asymptomatic and expressed appreciation for the clinical care received.

## 3. Discussion

### 3.1. Cardiac mechanisms

CO is a colorless, odorless gas commonly produced during combustion. Globally, ACOP is prevalent and results in tens of thousands of deaths annually.^[3]^ Oxygen therapy constitutes the cornerstone of treatment, functioning primarily by increasing the arterial oxygen partial pressure, which accelerates the dissociation of CO from hemoglobin. Currently, oxygen therapy for ACOP principally comprises 100% normobaric oxygen and HBOT.^[4]^ HBOT reduces the half-life of CO in the blood by more than half, from 74 minutes (on normobaric oxygen) to just 20 minutes.^[5–7]^ Moreover, studies have demonstrated that HBOT not only improves neurological function and reduces the incidence of neurological sequelae but may also enhance the prognosis of patients with ACOP by modulating inflammatory responses and promoting angiogenesis.^[8–10]^

APE associated with HBOT is a rare complication potentially linked to pathophysiological alterations in the cardiovascular system. Existing literature indicates that this complication predominantly occurs in patients with underlying heart disease, including heart failure with reduced ejection fraction (HFrEF) and heart failure with preserved ejection fraction (HFpEF) accompanied by impaired diastolic function.^[11–13]^ From a pathophysiological perspective, HBOT may affect cardiac function via a dual mechanism. First, the hyperoxic environment increases systemic vascular resistance, thereby significantly elevating cardiac afterload.^[14]^ Second, oxygen radical-mediated consumption of endothelium-derived nitric oxide can reduce left ventricular diastolic compliance and subsequently diminish left ventricular output.^[15,16]^ When the right heart maintains relatively normal output under sustained hyperoxic exposure, whereas the left heart exhibits impaired ejection, an imbalance in cardiac output ensues.^[14,17]^ This uncoupling of right and left ventricular function, particularly in patients with compromised ventricular-arterial coupling, may ultimately lead to pulmonary circulatory congestion and the clinical presentation of APE.

### 3.2. Noncardiac factors

In this patient, both echocardiographic and electrocardiographic assessments revealed normal findings, and no other overt etiologies of pulmonary edema (e.g., infection or allergic reaction) were detected. Although troponin I levels were mildly elevated (0.38 ng/mL) – a finding consistent with possible myocardial injury, presumably secondary to carbon monoxide poisoning – the absence of other cardiac abnormalities supports the predominance of noncardiac mechanisms. Therefore, other potential contributing factors warrant consideration. According to previous studies,^[18,19]^ CO poisoning and ischemia-hypoxia conditions may affect pulmonary capillary permeability, thereby increasing extravasation of intravascular fluids and leading to interstitial edema. Notably, the onset of symptoms occurred during the decompression phase of HBOT. The underlying mechanism may involve incomplete pulmonary tissue repair, whereby the decompression process induces vasodilation and increased permeability, whereas a reduction in airway pressure results in relatively higher capillary hydrostatic pressure. Together, these factors create a pressure gradient that synergistically promotes extravasation of intravascular fluid, ultimately triggering APE.

Mediastinal emphysema, subcutaneous emphysema, and mild pneumothorax were observed on CT images. These findings are mechanistically linked to an abrupt increase in alveolar pressure induced by severe coughing, which subsequently damages the alveolar–capillary barrier. During episodes of intense coughing, the sharp increase in alveolar pressure may exceed tissue tolerance, allowing gas to escape through a compromised alveolar basement membrane into the pulmonary interstitium. The gas then migrates along the pulmonary vascular sheaths toward the mediastinum and ultimately diffuses into the subcutaneous tissues and pleural cavity, resulting in mediastinal emphysema, subcutaneous emphysema, and pneumothorax.^[20]^

### 3.3. Management considerations

The management of APE is primarily supportive, with emphasis on improving oxygenation and reducing pulmonary edema. The decision to perform endotracheal intubation is contingent on the patient’s ability to maintain effective oxygenation. In this case, the patient was unable to sustain oxygenation and exhibited shallow, rapid breathing, indicative of early acute respiratory distress syndrome (ARDS). Consequently, we proceeded with endotracheal intubation and implemented lung-protective ventilation. Given the existing alveolar damage, positive end-expiratory pressure was maintained at 5 cm H_2_O. Furthermore, a targeted pharmacotherapeutic regimen was implemented, consisting of intravenous furosemide 20 mg, methylprednisolone 40 mg, and diprophylline 500 mg. The use of glucocorticoids remains controversial in the management of respiratory distress syndrome. Although early administration may mitigate inflammatory responses, current guidelines generally discourage their routine use due to associated infection risks.^[21]^ Nonetheless, judicious application during the early phase of the disease can help modulate excessive inflammation through inhibition of inflammatory mediator release and stabilization of cell membrane phospholipid structures. This therapeutic approach reduces tissue edema secondary to increased vascular permeability and may shorten the duration of mechanical ventilation by controlling the inflammatory cascade.^[22]^

## 4. Conclusion

In summary, although HBOT-induced APE is rare, it can lead to severe complications during ACOP treatment. Despite this risk, HBOT remains the most effective first-line clinical treatment for severe ACOP. Clinical implementation necessitates rigorous evaluation of treatment indications and potential risks coupled with enhanced monitoring and preventive measures throughout the treatment process.

## Author contributions

**Conceptualization:** Yu-Yang Chen.

**Formal analysis:** Zhi-Jian Gu.

**Investigation:** Zhi-Jian Gu.

**Methodology:** Yu-Yang Chen, Zhi-Jian Gu.

**Project administration:** Zhi-Jian Gu.

**Writing – original draft:** Min-Xue Yao, Yu-Yang Chen, Zhi-Jian Gu.

**Writing – review & editing:** Min-Xue Yao.
